# Potentially malignant oral lesions: clinicopathological correlations

**DOI:** 10.1590/S1679-45082016AO3578

**Published:** 2016

**Authors:** Haline Cunha de Medeiros Maia, Najara Alcântara Sampaio Pinto, Joabe dos Santos Pereira, Ana Miryam Costa de Medeiros, Éricka Janine Dantas da Silveira, Márcia Cristina da Costa Miguel

**Affiliations:** 1 Universidade Federal do Rio Grande do Norte, Natal, RN, Brazil.

**Keywords:** Leukoplakia/diagnosis, Erythroplasia, Cheilitis, Lichen planus, oral

## Abstract

**Objective:**

To determine the incidence of potentially malignant oral lesions, and evaluate and correlate their clinical and pathological aspects.

**Methods:**

The sample consisted of cases clinically diagnosed as oral leukoplakia, oral erythroplakia, erythroleukoplakia, actinic cheilitis, and oral lichen planus treated at a diagnostic center, between May 2012 and July 2013. Statistical tests were conducted adopting a significance level of 5% (p≤0.05).

**Results:**

Out of 340 patients, 106 (31.2%) had potentially malignant oral lesions; and 61 of these (17.9%) were submitted to biopsy. Actinic cheilitis was the most frequent lesion (37.5%) and the lower lip was the most affected site (49.6%). Among 106 patients in the sample, 48 (45.3%) reported nicotine consumption, 35 (33%) reported alcohol intake and 34 (32.1%) sun exposure while working. When clinical and histopathological diagnoses were compared, oral erythroplakia and atypical ulcer were the lesions that exhibited greater compatibility (100% each).

**Conclusion:**

In most cases, clinical and histopathological diagnoses were compatible. An association between the occurrence of erythroplakia, leukoplakia and erythroleukoplakia with smoking was observed. Similarly, an association between actinic cheilitis and sun exposure was noted. Erythroleukoplakia presented the highest malignancy grade in this study. Finally, dental surgeons should draw special attention to diagnosis of potentially malignant oral lesions, choose the best management, and control the lesions to avoid their malignant transformation.

## INTRODUCTION

Listed as one of the most frequent malignant neoplasms worldwide, oral cavity cancers are a significant public health problem.^1,2^ Most of these neoplasms stem from long-standing potentially malignant oral lesions (PMOL), partly due to the general public’s lack of knowledge about them and their associated etiologic factors.^1^

Precancerous lesions are defined as a morphologically altered tissue, in which cancer is more likely to occur than in its apparently normal counterpart, whereas a precancerous condition is an overall condition associated with a significantly higher risk of cancer.^3^ Some of these lesions have high potential for developing carcinoma, often regardless of the degree of epithelial dysplasia.^4^

The PMOL have varying levels of prevalence among the population, being often associated with environmental and behavioral differences, which influence exposure to etiological factors.^5^ Many of the agents associated with the development of oral cancer are also involved in the development of potentially malignant lesions, such as chronic exposure to UV radiation, alcoholism, smoking, nutritional deficiency, genetic inheritance^6^ and HPV infection.^7^

Leukoplakia, erythroplakia, actinic cheilitis and oral lichen planus are among the most commonly found precancerous oral lesions. Oral leukoplakia is a predominantly white lesion that may be of smooth, rough or warty texture.^3^ Oral erythroplakia, in turn, refers to a red macular or plaque-like lesion.^8^ Actinic cheilitis affects the lower lip in 95% of cases and appears as dryness, erosion and loss of definition in the lip contour.^9^ Oral lichen planus is of unknown etiology, in which an immune response attacks the lining epithelium. Several prospective and retrospective studies reported malignant transformation rates of oral lichen planus ranging from 0 to 9%.^10^

Once malignancy is confirmed, epidermoid carcinoma is the most common diagnosis - an invasive neoplasm of the squamous epithelium with varying degrees of differentiation, and propensity to early and extensive lymphatic metastases.^11^ Treatment usually consists of surgery and radiation therapy, but significant rates of local relapse are not infrequent.^12^ Several studies addressed investigations of malignant transformations of PMOL.^1,13^

Given their risk of undergoing malignant transformation, studies are required to assess the prevalence of these lesions in the population, in addition to demonstrate indicators to help health professionals recognize them.

## OBJECTIVE

To assess the incidence of potentially malignant oral lesions and to evaluate and correlate their clinical and pathological aspects.

## METHODS

A retrospective cross-sectional study, with a population comprised of all charts of patients with suspected PMOL, and with a sample comprised of cases with clinical diagnosis of oral leukoplakia, oral erythroplakia, erythroleukoplakia, actinic cheilitis, atypical ulcerated lesion and oral lichen planus, treated at the Oral Diagnosis Center, at the *Universidade Federal do Rio Grande do Norte*, Natal (RN), between May 2012 and July 2013. This research was submitted to and approved by the Research Ethics Committee, CAAE: 12130012.8.0000.5537 of the aforementioned university, opinion number 246.779, having met the requirements of resolution 466/12 of the National Health Council and of the Declaration of Helsinki. All patients signed an informed consent form for human experimentation.

Data was collected on a form specifically created for this research, containing patient information, including name, age, risk factors (smoking, drinking, and working in the sun), and lesion-related data, such as clinical characteristics, location, clinical and histopathological diagnoses, and management adopted by the dental surgeon after making diagnosis of the lesion. Forms were cross-checked to identify possible correlations in the data collected.

The results were organized into a database using the Statistical Package for the Social Sciences (SPSS) software, version 20.0. They were then submitted to a χ^2^ test for association of variables. A significance level of 5% (p≤0.05) was adopted.

## RESULTS

According to the data collected, 340 patients visited the diagnostic center between May 2012 and July 2013. PMOL were found in 106 of these patients, and 66 (62.3%) were male. Most patients with PMOL were aged 51-60 years (27; 25.5%), mean age of 56.09 years (minimum/maximum: 21/89). Most patients were white (66; 62.3%); the remaining (n=40; 37.7%) were mulatto and black. Sixty-one of 106 patients with PMOL were biopsied, three individuals had two lesions each, totaling up 64 biopsies.

In terms of sites affected, there were 60 lesions (49.6%) on the lower lip, 16 (13.2%) on the oral mucosa, 13 (10.7%) on the tongue, 11 (9.1%) on the alveolar ridge, 7 (5.8%) on the soft palate, 6 (5%) on the hard palate, 4 (3.3%) in the retromolar trigone, 2 (1.7%) on the upper lip and 2 (1.7 %) on the oral floor; in some cases, the same lesion would spread across two or more sites.

As for risk factors, 48 patients reported being current or former smokers, 35 reported drinking alcohol and 34 reported working under direct sunlight. Risk factors were associated to the patients’ gender and race (no reported data) and correlations between gender and consumption of alcohol, gender and sun exposure, and race and smoking were statistically significant (p<0.05).

A correlation was made between the clinical diagnosis and risk factors of each patient (Table 1).

**Table 1:** Clinical diagnosis and risk factors

Clinical diagnosis	Risk factors					

	Smoking		Drinking		Exposure to sunlight	
		
	No	Yes	No	Yes	No	Yes
	n (%)	n (%)	n (%)	n (%)	n (%)	n (%)
Actinic cheilitis	38 (64.4)	15 (30)	37 (50.7)	16 (44.4)	24 (32.4)	29 (82.9)
Oral lichen planus	5 (8.5)	1 (2)	2 (2.7)	4 (11.1)	6 (8.1)	0 (0)
Oral leukoplakia	8 (136)	19 (38)	20 (27.4)	7 (19.4)	24 (32.4)	3 (8.6)
Oral erythroplakia	2 (3.4)	4 (8)	3 (4.1)	3 (8.3)	5 (6.8)	1 (2.9)
Erythroleukoplakia	3 (5.1)	8 (16)	6 (8.2)	5 (13.9)	9 (12.2)	2 (5.7)
Atypical ulcerated lesion	1 (1.7)	3 (6)	3 (4.1)	1 (2.8)	4 (5.4)	0 (0)
Malignant mesenchymal lesion	1 (1.7)	0 (0)	1 (1.4)	0 (0)	1 (1.4)	0 (0)
Malignant vascular lesion	1 (1.7)	0 (0)	1 (1.4)	0 (0)	1 (1.4)	0 (0)

Evidence also pointed to a correlation between histopathological diagnosis and the patient’s risk factors, where some factors were more associated with the occurrence of certain lesions (Table 2).

**Table 2:** Histopathological diagnosis and risk factors

Histopathological diagnosis	Risk factors					

	Smoking		Drinking		Exposure to sunlight	
		
	No	Yes	No	Yes	No	Yes
	n (%)	n (%)	n (%)	n (%)	n (%)	n (%)
Oral epidermoid carcinoma	5 (14.7)	9 (30)	8 (14.7)	6 (30)	14 (29.8)	0 (0)
Oral epithelial dysplasia	16 (47.1)	11 (36.7)	16 (36.4)	11 (55.0)	16 (34)	11 (64.7)
No dysplasia	12 (35.3)	8 (26.7)	17 (38.6)	3 (15)	14 (29.8)	6 (35.3)
Oral lichen planus	1 (2.9)	2 (6.7)	3 (6.8)	0 (0)	3 (6.4)	0 (0)

Among patients with lesions, 45 cases were not biopsied and most were found to be actinic cheilitis (29; 64.4%). The 64 cases that underwent biopsy were clinically diagnosed as oral leukoplakia (20; 31.2%), erythroplakia (4; 6.2%), erythroleukoplakia (8, 12.5%), actinic cheilitis (24; 37.5%), oral lichen planus (3, 4.7%), atypical ulcerated lesion (4; 6.2%) and malignant mesenchymal lesion (1; 1.6%). In terms of compatibility between clinical and histopathological diagnoses, 14 lesions (21.9%) were histologically not compatible with the clinical diagnosis (Tables 3 and 4).

**Table 3:** Clinical diagnosis and histopathology

Clinical diagnosis	Histopathological diagnosis			

	OEC	No dysplasia	Oral epithelial dysplasia	Oral lichen planus
	n (%)	n (%)	n (%)	n (%)
Actinic cheilitis	1 (7.1)	6 (30)	15 (55.6)	2 (66.7)
Oral lichen planus	0 (0)	1 (5)	1 (3.7)	1 (33.3)
Oral leukoplakia	3 (21.4)	10 (50)	7 (25.9)	0 (0)
Oral erythroplakia	2 (14.3)	0 (0)	2 (7.4)	0 (0)
Erythroleukoplakia	3 (21.4)	3 (15)	2 (7.4)	0 (0)
Atypical ulcerated lesion	4 (28.6)	0 (0)	0 (0)	0 (0)
Malignant mesenchymal lesion	1 (7.1)	0 (0)	0 (0)	0 (0)

Total	14 (100)	20 (100)	27 (100)	3 (100)

OEC: Oral epidermoid carcinoma.

**Table 4:** Compatibility between clinical and histopathological diagnosis

Clinical diagnosis	Compatible n (%)	Not compatible n (%)	Total n (%)
Actinic cheilitis	20 (83.3)	4 (16.7)	24 (100)
Oral lichen planus	1 (33.3)	2 (66.7)	3 (100)
Oral leukoplakia	16 (80)	4 (20)	20 (100)
Oral erythroplakia	4 (100)	0 (0)	4 (100)
Erythroleukoplakia	5 (62.5)	3 (37.5)	8 (100)
Atypical ulcerated lesion	4 (100)	0 (0)	4 (100)
Malignant mesenchymal lesion	0 (0)	1 (100)	1 (100)

The procedure adopted by oral surgeons was also taken into consideration. For actinic cheilitis, the most common procedures were the application of fludroxycortide, use of sunscreen and biopsy. Twenty-six patients were given conservative treatment with fludroxycortide alone and/or sunscreen; in 23 cases a biopsy was indicated (of which 11 received conservative treatment before being biopsied) and in 4 cases no record of the procedure was made by the oral surgeon. For cases of oral leukoplakia, 20 patients were referred for biopsy; in one case, no record of the procedure was found; in three cases, additional exams were requested (complete blood count and fasting glucose), and in another three cases, the patients were followed-up. Of six cases with clinical diagnosis of oral erythroplakia, five were referred for biopsy, whereas one clearly had traumatic origin thus no biopsy was required. All cases of erythroleukoplakia were referred for biopsy. As to oral lichen planus, a biopsy was requested in six cases; in one case, laser therapy was performed before referral for biopsy, and in another there was no record of the procedure adopted by the oral surgeon.

## DISCUSSION

The high number of PMOL patients in this study is accounted for the fact that oral surgeons from the entire state refer patients to the reference center when they find greater difficulty in making diagnosis.

In cases where the oral surgeon requested a biopsy, 64.7% of patients failed to be at the surgery department to undergo the procedure. This is a significant amount, since a biopsy is the most accurate way to confirm a clinical diagnosis. In some cases, this can be explained by the fact that people are still afraid to learn they have cancer, and prefer to remain unaware of the diagnosis and possible treatments.^14^

The most affected sites were lower lips and oral mucosa. In a study by Bokor-Bratie et al.,^4^ lesions were more frequent in oral mucosa and alveolar ridge. According to a study by Silveira et al.,^15^ the most common sites were the lower lip and palate. Sites vary according to the type of lesion most commonly found in patients and the geographical region of the world where the study takes place, since some environmental and behavioral conditions are considered risk factors for certain lesions studied.^5^

The study indicated a higher occurrence of actinic cheilitis followed by oral leukoplakia, which differs from the findings of some investigations that reported leukoplakia as the most common and prevalent form of potentially malignant lesions.^16-18^ This can be explained by the fact that, according to the *Instituto de Pesquisas Espaciais*, in São José dos Campos, Brazil, in the city of Natal, the UV index is considered high during most of the year,^19^ and the main activity in the rural cities of the state of Rio Grande do Norte is agriculture, which exposes individuals to radiation.^20^

Among cases of oral leukoplakia, 44.4% of patients were white, contradicting what was demonstrated in studies by Silverman et al.^13^ and by Haas, Jr. et al.,^17^ where 97% and 80.8%, respectively, of patients with oral leukoplakia were white. This difference could be explained by the limited size of our sample and diversified ethnicity. As reported in other studies, females were more numerous.^1,15^ The mean age in our study was similar to that found by Silverman et al.^13^ (54 years) and Haas, Jr. et al.^17^ (55.9 years).

In the present study, oral leukoplakia was most frequently found in the oral mucosa and alveolar ridge, in line with the findings of other authors,^13,16,21^ who identified the oral mucosa as the site most commonly affected by these lesions. For Lapthanasupkul et al.,^21^ the location of lesions is related to the patient’s habits: smokers have a higher probability of developing lesions in the oral mucosa.

According to Pereira et al.^22^ oral erythroplakia affects both genders with higher incidence in men, and is more common among people aged between 40 and 60 years. This corroborates the results of the current study, where 66.7% of patients were male and the mean age was 65 years. Lapthanasupkul et al.^21^ described the palate as the most affected site. In our study, 22.2% of erythroplakias were found in the soft palate and 11.1% in the hard palate. Unlike the report by Reichart et al.^,23^ who stated oral erythroplakia is most prevalent among white individuals, our study indicates higher prevalence among patients of different races.

Actinic cheilitis was significantly more frequent in white males, confirming the findings of other authors.^9,24^ Moreover, in our study all cases of actinic cheilitis were located in the lower lip, in line with findings from literature.^9,25^

Oral lichen planus was prevalent among males, with a mean age of 45.8 years, diverging from the findings of several other studies,^26-28^ where this type of lesion mostly affected women. This is explained by the fact that there were only six cases of oral lichen planus in this study, which does not represent the general population. The literature demonstrates that lichen planus shows a predilection for the oral mucosa, which was also observed in our findings. It is believed that this preference for the oral mucosa is due to thickness of the epithelium and its degree of keratinization. Hence, the histopathological changes observed in oral lichen planus result in clinical manifestations in the oral mucosa more than in other mucosae.^28^

In terms of risk factors, there was a significant correlation between the consumption of nicotine and erythroplakia, leukoplakia and erythroleukoplakia. According to Neville et al.,^29^ tobacco is a potent carcinogen and, along with chronic alcoholism, is the most important risk factor for the development of head and neck cancer. Some studies showed that the etiology of erythroplakia may be associated with alcohol and tobacco abuse; deficiency of beta-carotene, vitamin C and E; carcinogens; viral infections; and genetic and hereditary factors.^6^

A comparison between the clinical and histopathological diagnosis resulted in a rate of confirmation of 78.1%; there was more agreement in atypical ulcerated lesions and oral erythroplakia. In oral erythroplakia, 50% of cases were diagnosed as oral epidermoid carcinoma and 50% as mild epithelial dysplasia. Histopathology of the lesion may indicate mild or moderate epithelial dysplasia, severe dysplasia, or carcinoma *in situ*.^23^

On the other hand, lesions that yielded the lowest agreement between clinical and histopathological diagnoses were malignant mesenchymal lesions, with only one lesion, and two cases of oral lichen planus, diagnosed as mild epithelial dysplasia and hyperkeratosis, incompatible with classic histological diagnosis.^30^ In a study with patients clinically diagnosed with oral lichen planus, 72% were confirmed by histopathological analysis; another 21% were diagnosed with frictional keratosis and 7% with epithelial dysplasia,^31^ leading to a suspicion that some cases of epithelial dysplasia may be clinically mistaken for oral lichen planus.

Oral leukoplakia reached 80% of coincidence between clinical and histopathological diagnoses. Considering only compatible lesions, 43.8% were histologically identified as mild, moderate and severe epithelial dysplasia. A study of leukoplakias and erythroplakias demonstrated the presence of some degree of epithelial dysplasia in 71% of surgically treated lesions and in 12% of not surgically treated lesions. This study indicated that the features of a lesion, such as clinical type, borders, site, size and patient’s smoking habits are important factors in determining the potential malignancy of the lesion.^32^

Histopathological exams diagnosed 14 cases of oral epidermoid carcinoma - most from clinically diagnosed lesions, such as atypical ulceration, leukoplakia or erythroleukoplakia. The latter, in spite of being less frequent, has high potential for malignancy. Oral erythroplakia, on the other hand, showed a potential for malignancy of 50% in our study, whereas literature demonstrates that up to 90% of these lesions are potentially malignant.^8^ The main purpose of recognizing PMOLs is to prevent their malignant transformation through the adequate intervention.^32^

As for the different management adopted by oral surgeons, fludroxycortide was exclusively used to treat actinic cheilitis in 12 cases, and was efficient in lesion regression. Fludroxycortide acts like other steroids, by decreasing and preventing tissue responses to inflammatory processes, and by reducing inflammation symptoms.^18^ The use of sunscreens as an effective form of protection has been widely discussed in the literature and recommended to prevent all types of skin cancers.^20,25^

## CONCLUSION

This study features a higher amount of actinic cheilitis cases, which is explained by the setting where the research was conducted. There was significant correlation between clinical and histopathological diagnoses, and erythroplakia and oral epidermoid carcinoma accounted for the highest percentages of confirmed cases. Erythroleukoplakia and oral leukoplakia were found to have the highest degree of malignancy among the lesions studied. We also found a correlation between exposure to carcinogens and the development of potentially malignant oral lesions. Therefore, oral surgeons are implicitly responsible for the early diagnosis of potentially malignant oral lesions, and for proper follow-up and treatment of each lesion. Studies like this contribute to the dissemination of knowledge, and help professionals make good decisions when facing the possible diagnosis of a potentially malignant oral lesion.
