# Supplementary material for: Potentially malignant oral lesions: clinicopathological correlations
Source: Einstein (Sao Paulo). 2016 Jan-Mar;14(1):35–40. doi: 10.1590/S1679-45082016AO3578 (PMC4872915; doi:10.1590/S1679-45082016AO3578)
Supplement: Translation

# Translation: Lesões orais potencialmente malignas: correlações clínico-patológicas

**Authors:** Haline Cunha de Medeiros Maia, Najara Alcântara Sampaio Pinto, Joabe dos Santos Pereira, Ana Miryam Costa de Medeiros, Éricka Janine Dantas da Silveira, Márcia Cristina da Costa Miguel

**Affiliations:** 1 Universidade Federal do Rio Grande do Norte, Natal, RN, Brasil.

**Keywords:** Leucoplasia/diagnóstico, Eritroplasia, Queilite, Líquen plano bucal

## Abstract

**Objetivo:**

Verificar a incidência das lesões orais potencialmente malignas, e avaliar e correlacionar seus aspectos clínico-patológicos.

**Métodos:**

A amostra foi composta pelos casos diagnosticados clinicamente como leucoplasia oral, eritroplasia oral, eritroleucoplasia, queilite actínica e líquen plano oral em um serviço de diagnóstico no período entre maio de 2012 e julho de 2013. Foram realizados testes estatísticos adotando-se nível de significância de 5% (p≤0,05).

**Resultados:**

Dos 340 pacientes examinados, 106 (31,2%) se apresentaram com lesões orais potencialmente malignas; destes, 61 (17,9%) biópsias foram realizadas. A lesão mais frequente foi a queilite actínica (37,5%), e o sítio anatômico mais acometido foi o lábio inferior (49,6%). Entre os 106 pacientes da amostra, 48 (45,3%) relataram consumo de nicotina, 35 (33%) ingeriam bebidas alcoólicas e 34 (32,1%) trabalhavam expostos ao sol. Comparando o diagnóstico clínico com o histopatológico, as lesões que apresentaram maior compatibilidade foram a eritroplasia oral e a lesão ulcerada atípica (100% ambas).

**Conclusão:**

Na maioria dos casos, houve compatibilidade do diagnóstico clínico com o histopatológico. Notou-se correlação entre a ocorrência de eritroplasia, leucoplasia e eritroleucoplasia com o hábito de fumar, e entre a queilite actínica com a exposição solar. A eritroleucoplasia foi a lesão que demonstrou maior grau de malignização neste estudo. Diante do exposto, recomenda-se maior atenção por parte dos cirurgiões-dentistas ao diagnóstico das lesões orais potencialmente malignas, para aplicação da melhor conduta e controle da lesão, impedindo sua transformação maligna.

## INTRODUÇÃO

Os cânceres da cavidade oral representam um importante problema de saúde pública, sendo relacionados como uma das neoplasias malignas mais frequentes em todo o mundo.^1,2^ A maioria dessas neoplasias é derivada de lesões orais potencialmente malignas (LOPM) existentes por um longo período de tempo, em parte por causa do desconhecimento da população sobre essas lesões e os fatores etiológicos a elas associados.^1^

As lesões pré-cancerosas são definidas como um tecido morfologicamente alterado, em que o câncer é mais provável de ocorrer que sua contraparte aparentemente normal, enquanto uma condição pré-cancerosa é definida como um estado generalizado associado a um risco significativamente aumentado de câncer.^3^ Algumas dessas lesões exibem um alto potencial de desenvolver um carcinoma, muitas vezes independentemente do grau de displasia epitelial.^4^

As LOPM apresentam prevalências diferentes na população, muitas vezes associadas com diferenças ambientais e de comportamento, o que influencia na exposição aos fatores etiológicos.^5^ Muitos dos agentes associados ao desenvolvimento do câncer de boca também estão envolvidos no surgimento de lesões potencialmente malignas, como exposição crônica à radiação ultravioleta, alcoolismo, tabagismo, deficiência nutricional, herança genética^6^ e infecção por HPV.^7^

Dentre as lesões orais pré-cancerosas, podem ser citadas como mais comuns a leucoplasia, a eritroplasia, a queilite actínica e o líquen plano oral. A leucoplasia oral é uma lesão predominantemente branca, que pode apresentar-se lisa, rugosa ou verrucosa.^3^ A eritroplasia oral, por sua vez, refere-se à lesão macular ou em placa, de coloração vermelha.^8^ A queilite actínica afeta o lábio inferior em 95% dos casos e se manifesta por ressecamento, erosões e perda do limite do lábio.^9^ O líquen plano oral possui etiologia desconhecida, havendo uma resposta imune com agressão ao epitélio de revestimento. Vários estudos prospectivos e retrospectivos têm relatado taxas de transformação maligna do líquen plano oral, que variam de 0 a 9%.^10^

Quando constatada a transformação maligna dessas lesões citadas, o diagnóstico mais frequente é de carcinoma epidermoide, neoplasia invasiva do epitélio escamoso com vários graus de diferenciação, e propensão para metástases linfáticas precoces e extensas.^11^ O tratamento geralmente consiste em cirurgia e radioterapia, mas ainda são observadas importantes taxas de recidivas locais.^12^ Ao longo do tempo, vários estudos se desenvolvem para tratar da ocorrência de transformação maligna nas LOPM.^1,13^

Considerando o risco de transformação maligna dessas lesões, são necessários estudos que verifiquem sua prevalência na população, bem como indicadores de reconhecimento correto pelos profissionais de saúde.

## OBJETIVO

Verificar a incidência das lesões orais potencialmente malignas, e avaliar e correlacionar seus aspectos clínico-patológicos.

## MÉTODOS

Estudo retrospectivo do tipo transversal, com população constituída por todos os prontuários de pacientes com suspeita de LOPM, tendo como amostra os casos com diagnóstico clínico de leucoplasia oral, eritroplasia oral, eritroleucoplasia, queilite actínica, lesão ulcerada atípica e líquen plano oral atendidos no serviço de Diagnóstico Oral da Universidade Federal do Rio Grande do Norte, Natal (RN), no período de maio de 2012 a julho de 2013. Esta pesquisa foi submetida e aprovada pelo Comitê de Ética em Pesquisa, CAAE: 12130012.8.0000.5537 da mesma universidade, sob parecer número 246.779, e cumpriu os requisitos da resolução 466/12 do Conselho Nacional de Saúde e da Declaração de Helsinque. Foi obtido consentimento informado para experimentação com seres humanos de todos os pacientes.

Na coleta dos dados, foi utilizada uma ficha especialmente criada para a pesquisa, contendo dados do paciente, como nome, idade, fatores de risco (fumar, beber e trabalhar exposto ao sol), e dados da lesão, como características clínicas, localização, diagnóstico clínico, diagnóstico histopatológico da lesão e conduta do cirurgião-dentista diante do diagnóstico da lesão. As informações coletadas das fichas clínicas foram correlacionadas com o objetivo de verificar a existência de possíveis associações entre elas.

Os resultados obtidos foram organizados em um banco de dados com o auxílio do programa *Statistical Package for the Social Sciences* (SPSS) na versão 20.0. Depois, os dados foram submetidos ao teste χ2 para associação das variáveis. Foi adotado nível de significância de 5% (p≤0,05).

## RESULTADOS

De acordo com a coleta de dados realizada, 340 pacientes compareceram ao serviço de diagnóstico entre maio de 2012 a julho de 2013. Destes, 106 se apresentaram clinicamente com LOPM, sendo 66 (62,3%) deles do gênero masculino. Entre os indivíduos com LOPM, a faixa etária mais frequente foi de 51 a 60 anos (27; 25,5%), com média de 56,09 anos (mínima/máxima: 21/89). A maior parte dos pacientes pertencia à raça branca (66; 62,3%); os demais (n=40; 37,7%) eram pardos e negros. Dos 106 pacientes portadores de LPOM durante esse período, 61 realizaram biópsia, sendo que, destes, 3 pacientes apresentaram 2 lesões cada, totalizando 64 biópsias realizadas.

Em relação aos sítios acometidos, verificaram-se 60 lesões (49,6%) em lábio inferior, 16 (13,2%) em mucosa jugal, 13 (10,7%) em língua, 11 (9,1%) em rebordo alveolar, 7 (5,8%) em palato mole, 6 (5%) em palato duro, 4 (3,3%) em trígono retromolar, 2 (1,7%) em lábio superior e 2 (1,7%) em assoalho bucal, sendo que, em alguns casos, uma mesma lesão se estendia para 2 ou mais localizações.

Quanto aos fatores de risco, 48 pacientes relataram ser fumantes ou ex-fumantes, 35 relataram consumo de bebida alcoólica e 34 relataram a condição de trabalho com exposição a raios solares. Os fatores de risco foram associados ao gênero e à raça dos pacientes (dados não apresentados), sendo estatisticamente significativas (p<0,05) as correlações entre gênero e consumo de álcool, gênero e exposição solar e raça e fumo.

Foi realizada a correlação entre o diagnóstico clínico e os fatores de risco apresentados pelos pacientes (Tabela 1).

**Tabela 1:** Diagnósticos clínicos e fatores de risco

Diagnóstico clínico	Fatores de risco					

	Consumo de nicotina		Consumo de álcool		Exposição a raios solares	
		
	Não	Sim	Não	Sim	Não	Sim
	n (%)	n (%)	n (%)	n (%)	n (%)	n (%)
Queilite actínica	38 (64,4)	15 (30)	37 (50,7)	16 (44,4)	24 (32,4)	29 (82,9)
Líquen plano oral	5 (8,5)	1 (2)	2 (2,7)	4 (11,1)	6 (8,1)	0 (0)
Leucoplasia oral	8 (136)	19 (38)	20 (27,4)	7 (19,4)	24 (32,4)	3 (8,6)
Eritroplasia oral	2 (3,4)	4 (8)	3 (4,1)	3 (8,3)	5 (6,8)	1 (2,9)
Eritroleucoplasia	3 (5,1)	8 (16)	6 (8,2)	5 (13,9)	9 (12,2)	2 (5,7)
Lesão ulcerada atípica	1 (1,7)	3 (6)	3 (4,1)	1 (2,8)	4 (5,4)	0 (0)
Lesão mesenquimal maligna	1 (1,7)	0 (0)	1 (1,4)	0 (0)	1 (1,4)	0 (0)
Lesão vascular maligna	1 (1,7)	0 (0)	1 (1,4)	0 (0)	1 (1,4)	0 (0)

Pôde-se verificar também a correlação do diagnóstico histopatológico com os fatores de risco dos pacientes, notando-se que alguns fatores estavam mais associados com a ocorrência de determinadas lesões (Tabela 2).

**Tabela 2:** Diagnóstico histopatológico e fatores de risco

Diagnóstico histopatológico	Fatores de risco					

	Consumo de nicotina		Consumo de álcool		Exposição a raios solares	
		
	Não	Sim	Não	Sim	Não	Sim
	n (%)	n (%)	n (%)	n (%)	n (%)	n (%)
Carcinoma epidermoide oral	5 (14,7)	9 (30)	8 (14,7)	6 (30)	14 (29,8)	0 (0)
Displasia epitelial oral	16 (47,1)	11 (36,7)	16 (36,4)	11 (55,0)	16 (34)	11 (64,7)
Sem displasia	12 (35,3)	8 (26,7)	17 (38,6)	3 (15)	14 (29,8)	6 (35,3)
Líquen plano oral	1 (2,9)	2 (6,7)	3 (6,8)	0 (0)	3 (6,4)	0 (0)

Dentre os pacientes com lesões, 45 casos não foram biopsiados, sendo a maioria dos casos de queilite actínica (29; 64,4%). Os 64 casos submetidos à biópsia foram diagnosticados clinicamente como leucoplasia oral (20; 31,2%), eritroplasia (4; 6,2%), eritroleucoplasia (8; 12,5%), queilite actínica (24; 37,5%), líquen plano oral (3; 4,7%), lesão ulcerada atípica (4; 6,2%) e lesão mesenquimal maligna (1; 1,6%). Em relação à compatibilidade entre diagnóstico clínico e histopatológico, 14 lesões (21,9%) não foram compatíveis histologicamente com o diagnóstico clínico (Tabela 3 e 4).

**Tabela 3:** Diagnóstico clínico e aspecto histopatológico

Diagnóstico clínico	Diagnóstico histopatológico			

	CEO	Sem displasia	Displasia epitelial oral	Líquen plano oral
	n (%)	n (%)	n (%)	n (%)
Queilite actínica	1 (7,1)	6 (30)	15 (55,6)	2 (66,7)
Líquen plano oral	0 (0)	1 (5)	1 (3,7)	1 (33,3)
Leucoplasia oral	3 (21,4)	10 (50)	7 (25,9)	0 (0)
Eritroplasia oral	2 (14,3)	0 (0)	2 (7,4)	0 (0)
Eritroleucoplasia	3 (21,4)	3 (15)	2 (7,4)	0 (0)
Lesão ulcerada atípica	4 (28,6)	0 (0)	0 (0)	0 (0)
Lesão mesenquimal maligna	1 (7,1)	0 (0)	0 (0)	0 (0)

Total	14 (100)	20 (100)	27 (100)	3 (100)

CEO: carcinoma epidermoide oral.

**Tabela 4:** Diagnóstico clínico e compatibilidade do diagnóstico histopatológico

Diagnóstico clínico	Compatível n (%)	Não compatível n (%)	Total n (%)
Queilite actínica	20 (83,3)	4 (16,7)	24 (100)
Líquen plano oral	1 (33,3)	2 (66,7)	3 (100)
Leucoplasia oral	16 (80)	4 (20)	20 (100)
Eritroplasia oral	4 (100)	0 (0)	4 (100)
Eritroleucoplasia	5 (62,5)	3 (37,5)	8 (100)
Lesão ulcerada atípica	4 (100)	0 (0)	4 (100)
Lesão mesenquimal maligna	0 (0)	1 (100)	1 (100)

Foi também observada a conduta dos cirurgiões-dentistas para o tratamento das lesões orais. Para a queilite actínica, as principais condutas foram aplicação de fludroxicortida, uso de protetor solar e biópsia. Em 26 casos, foi realizado um tratamento conservador com uso apenas de fludroxicortida e/ou protetor solar; em 23 casos, foi indicada a realização de biópsia (destes, 11 realizaram tratamento conservador antes de indicar a biópsia) e, em 4 casos, não foi registrada a conduta tomada pelo cirurgião-dentista. Nos casos de leucoplasia oral, 20 pacientes foram encaminhados para biópsia e, em 1 dos casos, o profissional não registrou a conduta indicada; em 3 casos, foram solicitados exames complementares (hemograma e glicemia em jejum) e, em outros 3, o paciente ficou em acompanhamento. De seis casos com diagnóstico clínico de eritroplasia oral, cinco foram encaminhados para biópsia; um não foi necessário, porque notou-se que a lesão era de origem traumática. Para os casos de eritroleucoplasia, todos os pacientes foram encaminhados para biópsia. Nos casos de líquen plano oral, foi indicada a realização de biópsia em seis casos; em um caso, foi realizada laserterapia antes do encaminhamento para biópsia e, em outro, não foi registrada a conduta do cirurgião-dentista.

## DISCUSSÃO

No presente estudo, o número elevado de pacientes com LOPM pode ser explicado pelo fato de os cirurgiões-dentistas de todo o Estado encaminharem para o centro de referência onde foi realizada esta pesquisa pacientes cujo diagnóstico apresenta algum grau de dificuldade maior.

Nos casos em que o cirurgião-dentista encaminhou o paciente para a realização de biópsia, 64,7% não compareceram ao setor cirúrgico para realizar o procedimento. Esse número é significativo, já que a biópsia é o meio mais seguro de confirmar o diagnóstico clínico. Em alguns casos, essa evasão é fruto do medo que as pessoas ainda possuem da descoberta de um possível câncer, o que faz com que as mesmas prefiram continuar sem saber o diagnóstico e o possível tratamento das lesões.^14^

Os sítios mais acometidos por essas lesões foram o lábio inferior e a mucosa jugal. Em estudo realizado por Bokor-Bratie et al.,^4^ as lesões se apresentaram mais em mucosa jugal e rebordo alveolar. Segundo Silveira et al.,^15^ os sítios mais frequentes em sua pesquisa foram lábio inferior e palato. Essa localização varia de acordo com o tipo de lesão mais encontrada nos pacientes e a região do mundo onde é realizada a pesquisa, uma vez que algumas condições ambientais e de comportamento são consideradas fatores de risco para determinadas lesões estudadas.^5^

Observou-se maior ocorrência de casos de queilite actínica, seguidos de leucoplasia oral, o que diverge de alguns estudos que relatam que, dentre as lesões potencialmente malignas, a leucoplasia é a mais comum e prevalente.^16-18^ Isso pode ser explicado pelo fato de Natal ser uma cidade onde o índice ultravioleta, de acordo com o Instituto de Pesquisas Espaciais de São José dos Campos, é considerado alto na maior parte do ano,^19^ e de a principal atividade nas cidades do interior do Rio Grande do Norte ser a agricultura, condição que expõe os indivíduos à radiação.^20^

Nos casos de leucoplasia oral, verificou-se que 44,4% dos pacientes pertenciam à raça branca, contradizendo o que pode ser visto nos trabalhos de Silverman et al.,^13^ em que 97% dos casos de leucoplasia oral eram em pacientes brancos, e Haas Jr. et al.,^17^ no qual 80,8% dos pacientes eram brancos. Tal diferença pode ser observada devido ao tamanho limitado da nossa amostra e da diferente etnia. Quanto ao gênero, assim como relatado em outros estudos, houve predominância do gênero feminino.^1,15^ Quanto à idade, a média apresentada em nosso estudo foi aproximada à obtida nos estudos de Silverman et al.^13^ (54 anos) e Haas Jr. et al.^17^ (55,9 anos).

Em nosso estudo, a leucoplasia oral apresentou predileção pela mucosa jugal e rebordo alveolar, resultado em consonância com os relatos de outros autores,^13,16,21^ que apresentaram a mucosa jugal como o local mais afetado por essas lesões. Para Lapthanasupkul et al.,^21^ a localização da lesão está relacionada ao hábito que o paciente apresenta: pacientes fumantes teriam maior predileção a manifestar a lesão em mucosa jugal.

Segundo Pereira et al.,^22^ a eritroplasia oral acomete ambos os gêneros, mas mostra maior incidência em homens, além de ser mais comum em pessoas com idade entre 40 e 60 anos, o que corrobora os resultados deste estudo, no qual 66,7% dos pacientes eram do gênero masculino e a média de idade foi de 65 anos. Para Lapthanasupkul et al.,^21^ o palato foi o sítio mais acometido, no nosso estudo, por sua vez, 22,2% das eritroplasias foram verificadas em palato mole e 11,1% em palato duro. Diferentemente do relato de Reichart et al.,^23^ que afirma que a raça mais prevalente acometida por eritroplasia oral em seu estudo foi a branca, no presente estudo foi verificada maior prevalência de pacientes de outras raças.

A queilite actínica foi consideravelmente mais frequente em homens, de cor branca, dado que é confirmado na literatura com o que foi visto por outros autores.^9,24^ Além disso, em nosso estudo, todos os casos de queilite actínica estavam localizados em lábio inferior, em consonância com os achados da literatura.^9,25^

Puderam ser notadas a prevalência do líquen plano oral no gênero masculino e a média de idade de 45,8 anos, divergindo quanto ao sexo mais acometido em vários estudos,^26-28^ nos quais essa lesão oral afetou mais mulheres. Isso se deveu ao fato de haver apenas seis casos de líquen plano oral no presente estudo, o que não representa com fidedignidade o acontece na população em geral. Observa-se na literatura que o líquen plano exibe predileção pela mucosa jugal, o que foi verificado também em nossos resultados. Acredita-se que a predileção do líquen plano oral pela mucosa jugal seja devido à espessura do epitélio e ao seu grau de ceratinização, pois isso possibilita que alterações histopatológicas observadas no líquen plano oral se reflitam clinicamente com mais facilidade na mucosa jugal do que em outras mucosas.^28^

Quanto aos fatores de risco apresentados pelos pacientes, houve relação considerável dos pacientes com eritroplasia, leucoplasia e eritroleucoplasia com o consumo de nicotina. De acordo com Neville et al.,^29^ o fumo do tabaco é um carcinógeno potente e, juntamente do alcoolismo crônico, torna-se o mais importante fator de risco para o desenvolvimento de câncer de cabeça e pescoço. Estudos revelam que a etiologia da eritroplasia pode estar associada a abuso de álcool e tabaco; deficiência de betacaroteno, vitamina C e E; agentes cancerígenos; infecções virais; e fatores genéticos e hereditários.^6^

Confrontando o diagnóstico clínico com o histopatológico, obteve-se índice de 78,1% de confirmação do diagnóstico, sendo as lesões mais coincidentes a lesão ulcerada atípica e a eritroplasia oral. Da eritroplasia oral, 50% foram diagnosticadas como carcinoma epidermoide oral e 50% como displasia epitelial leve. A histopatologia dessa lesão pode apresentar displasias epiteliais leves ou moderadas, displasias graves ou carcinomas *in situ*.^23^

Em contrapartida, as lesões que apresentaram menor coincidência entre o diagnóstico clínico e histopatológico foram a lesão mesenquimal maligna, com uma lesão apenas, e o líquen plano oral, obtendo como resultado de dois casos os diagnósticos de displasia epitelial leve e hiperceratose, incompatíveis com o diagnóstico histológico clássico.^30^ Em estudo realizado com pacientes diagnosticados clinicamente como líquen plano oral, 72% foram confirmados em análise histopatológica; os outros 21% foram diagnosticados com ceratose friccional e 7% com displasia epitelial,^31^ levando à suspeita de que alguns casos de displasias epiteliais podem ser confundidos clinicamente com líquen plano oral.

A leucoplasia oral demonstrou 80% de conformidade entre o diagnóstico clínico e o histopatológico. Considerando apenas as lesões compatíveis, 43,8% exibiram aspecto histológico de displasia epitelial nas graduações leve, moderada e severa. Estudo com leucoplasias e eritroplasias observou a presença de algum grau de displasia epitelial em 71% das lesões tratadas cirurgicamente e em 12% daquelas não tratadas cirurgicamente. Esse trabalho afirma que características como tipo clínico da lesão, demarcação, localização, tamanho e tabagismo do paciente são fatores importantes para o potencial de malignização da lesão.^32^

Ao exame histopatológico, foram diagnosticados 14 casos de carcinoma epidermoide oral − a maioria proveniente de lesões diagnosticadas clinicamente, como ulceração atípica, leucoplasia ou eritroleucoplasia. Esta última, apesar de se tratar de uma lesão menos comum, demonstrou alto potencial de malignização em nosso estudo. Já a eritroplasia oral apresentou nesse estudo um potencial de malignização de 50%, enquanto na literatura se verifica grau malignização em até 90% das lesões.^8^ O objetivo principal de identificar LOPM é impedir sua transformação maligna, realizando a intervenção adequada.^32^

Quanto à conduta dos cirurgiões-dentistas frente ao tratamento das lesões, a fludroxicortida foi usada exclusivamente no tratamento da queilite actínica em 12 casos e mostrou-se eficaz pela regressão da lesão. A fludroxicortida possui o mesmo mecanismo de ação de outros corticosteroides, contribuindo para a redução e a prevenção das respostas do tecido aos processos inflamatórios, e reduzindo os sintomas da inflamação.^18^ A utilização de fotoprotetores como forma efetiva de proteção tem sido amplamente discutida na literatura, sendo recomendada para prevenção de todas as neoplasias da pele.^20,25^

## CONCLUSÃO

Verificou-se, em nosso estudo, um maior número de casos de queilite actínica, fato explicado pelo local onde a pesquisa foi realizada. Houve relevante correlação entre os diagnósticos clínico e histopatológico, sendo a eritroplasia e o carcinoma epidermoide oral as lesões responsáveis pelas maiores porcentagens de casos confirmados. A eritroleucoplasia e a leucoplasia oral apresentaram o maior grau de malignização dentre as lesões estudadas. Observou-se também correlação entre a exposição à carcinógenos e a ocorrência das lesões orais potencialmente malignas. Diante disso, fica implícita a responsabilidade do cirurgião dentista em diagnosticar as lesões orais potencialmente malignas precocemente, e de realizar o acompanhamento e tratamento indicados para cada lesão. Pesquisas como esta contribuem para a divulgação do conhecimento e auxiliam na tomada de decisão dos profissionais que se encontram diante da suspeita de ocorrência de uma lesão oral potencialmente maligna.
